# Epidermal SIRT1 regulates inflammation, cell migration, and wound healing

**DOI:** 10.1038/s41598-017-14371-3

**Published:** 2017-10-26

**Authors:** Lei Qiang, Ashley Sample, Han Liu, Xiaoyang Wu, Yu-Ying He

**Affiliations:** 1 Department of Medicine, Section of Dermatology, University of Chicago, Chicago, IL 60637 USA; 2 State Key Laboratory of Natural Medicines, Jiangsu Key Laboratory of Carcinogenesis and Intervention, School of Basic Medicine and Clinical Pharmacy, China Pharmaceutical University, Nanjing, 211198 China; 3 Committee on Cancer Biology, University of Chicago, Chicago, IL 60637 USA; 4 Ben May Department of Cancer Research, University of Chicago, Chicago, IL 60637 USA

## Abstract

Sirtuins (SIRT1-7) are NAD-dependent proteins with the enzymatic activity of deacetylases and ADP ribosyltransferases. SIRT1 is the proto member of the proteins in the mammalian sirtuin family and plays multiple roles in aging and disease. Using mice with epidermis-specific SIRT1 deletion, we show that SIRT1 is required for efficient wound healing. SIRT1 deficiency in the epidermis inhibited the regeneration of both the epidermis and the dermal stroma. SIRT1 loss altered the production of many cytokines, inhibited the recruitment of macrophages, neutrophils, and mast cells, the recruitment and activation of fibroblasts, and angiogenesis in the granulation tissue. In keratinocytes, SIRT1 knockdown inhibited EMT, cell migration, and TGF-β signaling. For the first time, using skin-specific mouse model, we demonstrate that epidermal SIRT1 plays a crucial role in wound repair. These findings are novel in understanding how wound healing is regulated. Our findings provide *in vivo* and *in vitro* evidence that SIRT1 in the epidermis regulates cell migration, redox response, inflammation, epidermis re-epithelialization, granulation formation, and proper wound healing in mice.

## Introduction

Sirtuins (SIRT1-7) are NAD-dependent proteins with the enzymatic activity of deacetylases and ADP ribosyltransferases^1–5^. They are mammalian counterparts of the yeast silent information regulator 2 (Sir2). Since SIRT1 was first discovered about 15 years ago, there have been major breakthroughs in understanding the critical roles of sirtuins in physiology and pathology^1–5^. Sirtuins regulate a wide variety of proteins in the nucleus, cytosol and mitochondria. They are crucial regulators of tissue homeostasis and adaptation under metabolic, oxidative, or genotoxic stress. In recent advances, animal models have been used to illuminate the roles of sirtuins in DNA repair, telomere integrity, metabolism, survival, inflammation, cell differentiation and stem cell biology in many human diseases^1–7^. Based on the molecular and cellular targets identified, sirtuins are considered to have important roles in cancer and age-related diseases in the skin^8^ and other organs^6,9^.

Injury to adult tissues initiates a sophisticated repair process in order to restore the damaged body site. The molecular and cellular events in wound repair must be tightly regulated and synchronized to re-establish the integrity of the affected tissue and homeostasis in the whole organism. Defects in wound repair constitute a severe health problem that frequently affects aged individuals, patients with diabetes or immunosuppression, and patients who receive chemotherapy or radiotherapy^10^. These individual often develop painful, non-healing ulcers. One severe complication of non-healing ulcers is the malignant transformation and development of cancer in fibrotic tissue.

Cutaneous wound healing is a well-coordinated multistep process that involves inflammation, re-epithelialization, and maturation. The initial inflammatory response stimulates the release of growth factors and chemokines that initiate the re-epithelialization phase of wound repair^11,12^. The process of wound re-epithelialization requires efficient coordination of multiple events, including the formation of a provisional wound bed matrix, the proliferation and migration of keratinocytes into the wound, the differentiation of new epithelium into stratified epidermis, and the activation and migration of fibroblasts into the provisional matrix. However, it remains poorly understood how these processes are coordinated and regulated at the molecular, cellular and organismal levels.

Indeed, using mouse models and *in vitro* systems, recent studies have illustrated critical roles of sirtuin proteins, in particular SIRT1, in the skin stress response, homeostasis, and skin diseases^13^. We have recently shown that epidermal SIRT1 plays an important role in regulating DNA repair and skin carcinogenesis induced by UVB radiation^14,15^ and skin barrier integrity^16^. In primary human corneal epithelial cells, overexpression of SIRT1 promotes glucose-attenuated cell migration^17^. In mice, moderate global overexpression of SIRT1 showed a reduction in a number of aging-related changes and diseases such as cancer^18^. However, only one of the two SIRT1-transgenic mouse lines showed improved wound healing at an older age^18^. Therefore, the role of SIRT1 in the healing of skin wounds remains unclear. Here, using mice with epidermis-specific SIRT1 deletion, we show that SIRT1 is required for efficient wound healing. SIRT1 regulates epidermal re-epithelialization and generation of dermal granulation tissue as well as keratinocyte migration, cytokine expression, TGF-β signaling, and oxidative stress.

## Results

### Epidermis-specific deletion of SIRT1 inhibits wound healing

**Figure 1:**
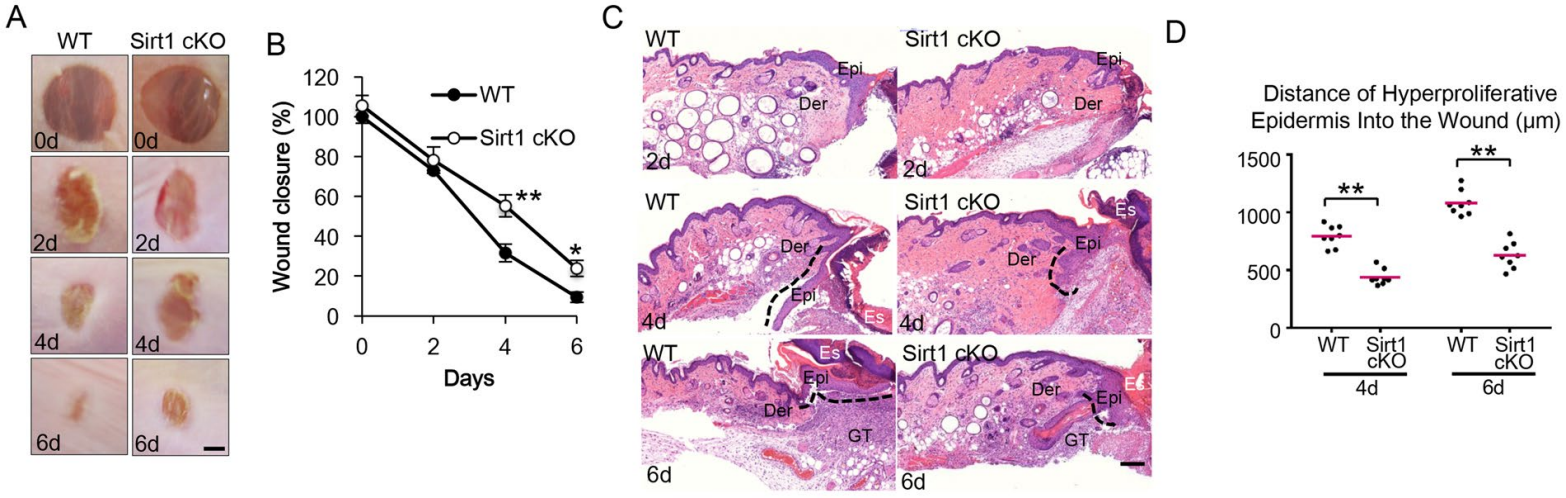
Epidermis-specific deletion of Sirt1 inhibits wound healing. (**A**) Macroscopic photographs of wound healing on day 0, 2, 4 and 6 in WT and Sirt1 cKO mice. 4 mm wounds were created on the backs of mice and wound closure was monitored. Scale bar: 1 mm. (**B**) Quantification of A (n = 8). (**C**) Wound healing as monitored by histological staining of skin sections at the wound edges 2, 4 and 6 days after injury in WT and Sirt1 cKO mice. Halves of wound sections on days 2, 4 and 6 are shown. Epi: epidermis; Der: dermis; Es: eschar. Black dotted lines denote the epidermal–dermal boundaries. Scale bar: 200 μm. (**D**) Quantification of the length of hyperproliferative epidermis generated at the indicated times after wounding. Data are shown as mean ± SD (n = 8). ***P < 0.001 compared with WT group (Student’s *t* test).

To determine the role of SIRT1 in skin wound healing, we first assessed the difference in wound repair between wild-type (WT) mice and mice with a SIRT1 deletion in their epidermis (SIRT1 cKO, conditional knockout), using full-thickness excisional wounds introduced to the dorsal skin of mice. As compared with WT mice, mice with epidermal SIRT1 deficiency showed a significant delay in wound closure (Fig. 1A and B). Histological analysis further confirmed delayed wound repair, granulation tissue formation, and re-epithelialization in SIRT1 cKO mice as compared with WT mice (Fig. 1C–E). These results indicate that SIRT1 deletion in the epidermis inhibits wound healing.

### Epidermis-specific deletion of SIRT1 inhibits angiogenesis in granulation tissue

**Figure 2:**
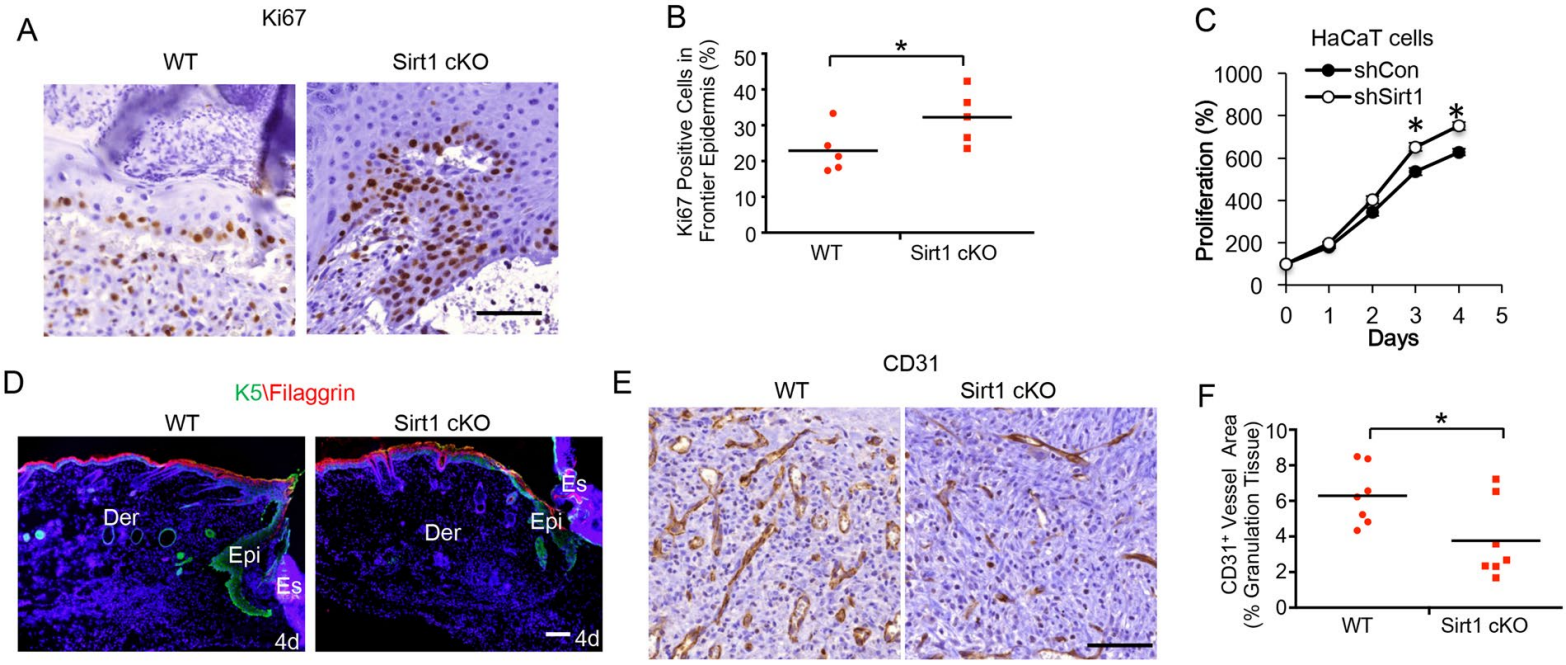
Epidermis-specific deletion of Sirt1 decreased angiogenesis in granulation tissue. (**A**) Immunohistochemical staining of Ki67 in wound sections on days 2, 4 and 6 in WT and Sirt1 cKO mice. Scale bar: 50 μm. (**B**) Quantification of ratio of Ki67 positive cells in regenerating epidermis (n = 5). (**C**) MTS assay of cell proliferation of HaCaT cells stably transfected with shCon and shSirt1. The results were obtained from three independent experiments (mean ± S.D., n = 3); *P < 0.05, compared with the shCon group). (**D**) Immunofluorescence staining of Filaggrin and K5 in wound sections on day 4 in WT and Sirt1 cKO mice. Scale bar: 200 μm. Epi: epidermis; Der: dermis; Es: eschar. (**E**) Immunohistochemical staining of CD31 in wound sections on days 2, 4 and 6 in WT and Sirt1 cKO mice. Scale bar: 200 μm. (**F**) Scatter plot and mean values of CD31 positive vessel number per high-power field (HPF; ×400 magnification) in wound sections on days 2, 4 and 6 in WT and Sirt1 cKO mice (n = 7) (HPF; ×40 magnification). *P < 0.05, as compared with the WT group.

To determine the mechanism of epidermal SIRT1 regulation of wound healing, we assessed the role of SIRT1 in keratinocyte proliferation, which is important for wound healing, by analyzing the number of Ki67-positive cells, as an indicator for cell proliferation and regeneration after wounding. As compared with WT mice, there were more Ki67-positive cells in the frontier epidermis of the wound area in mice with epidermis-specific SIRT1 deletion (Fig. 2A and B). In the human keratinocyte cell line HaCaT *in vitro*, SIRT1 knockdown increased cell proliferation (Fig. 2C). Next, we investigated the role of SIRT1 in keratinocyte differentiation, which is also important for wound healing, by monitoring the terminal differentiation marker filaggrin and basal layer marker Keratin (K5). We did not detect filaggrin in the newly generated epidermis of either WT or SIRT1 cKO mice (Fig. 2D). K5 was detected in the basal layers of the newly generated epidermis in WT mice, while it was reduced in the counterpart of SIRT1 cKO mice (Fig. 2D). We also investigated the angiogenesis in granulation tissue by monitoring the presence of endothelial cell marker CD31. As compared with WT mice, SIRT1 cKO mice exhibited reduced CD31-positive blood vessels in the granulation tissue (Fig. 2E and F). These data indicate that epidermis-specific SIRT1 deletion decreases K5-positive keratinocytes in the regenerated epidermis and angiogenesis in granulation tissue, while it does not affect keratinocyte proliferation.

### Epidermis-specific deletion of SIRT1 inhibits recruitment of macrophages, neutrophils, and mast cells

**Figure 3:**
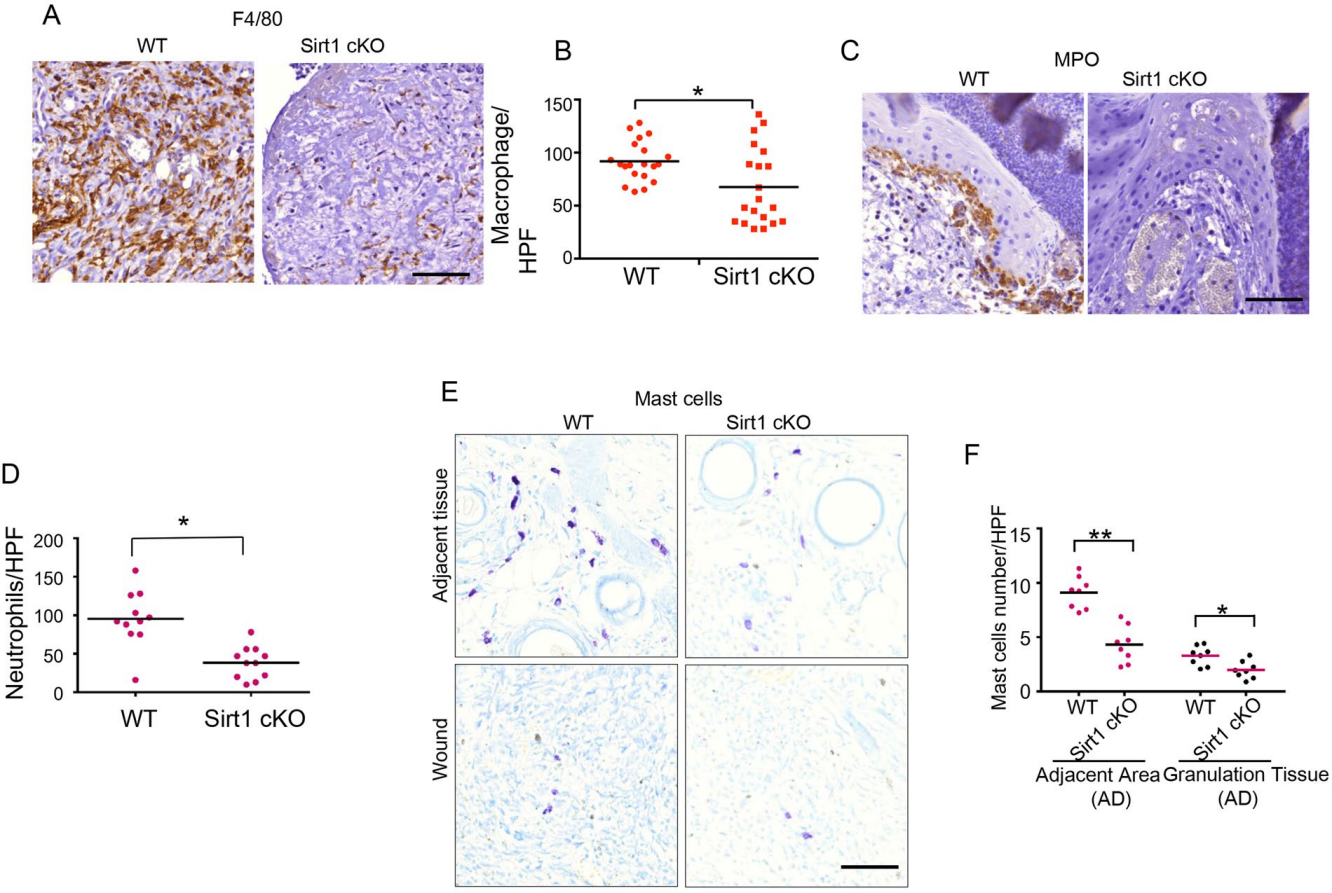
Epidermis-specific deletion of Sirt1 inhibits recruitment of macrophages, neutrophils, and mast cells. (**A**) Immunohistochemical staining of F4/80 in wound sections on days 2, 4 and 6 in WT and Sirt1 cKO mice. Blue dotted lines denote the granulation tissue. Scale bar: 200 μm. (**B**) Quantification of A (n = 20). (**C**) Immunohistochemical staining of MPO in wound sections on days 2 and 4 in WT and Sirt1 cKO mice. Scale bar: 50 μm for upper and lower panels, respectively. (**D**) Scatter plot and mean values of neutrophil number per high-power field (HPF; × 400 magnification) in wound sections from C (n = 11). (**E**) Toluidine blue staining of mast cells in wound sections on day 6 in WT and Sirt1 cKO mice. Scale bar: 50 μm. (**F**) Scatter plot and mean values of master cell number per high-power field (HPF; ×400 magnification) in wound sections on day 6 in WT and Sirt1 cKO mice (n = 8).

Wound-induced inflammation is the first stage in the wound healing process and has been demonstrated to play important roles in wound repair^11,12^. To determine the role of epidermal SIRT1 deficiency in wound-induced inflammation, we analyzed the difference in the recruitment of different inflammatory cells following wounding, including macrophages, neutrophils, and mast cells. Macrophages have multiple functions in wound repair, including host defense, promotion and resolution of inflammation, removal of apoptotic cells, and support for cell proliferation and tissue restoration following wounding^19^. At Day 6 in WT mice, F4/80-positive macrophages had been recruited to the granulation tissue of the wound, while epidermis-specific deletion of SIRT1 decreased macrophage recruitment to the wound area (Fig. 3A and B). In addition, neutrophils are the first nucleated immune cells to infiltrate into a wound, acting as a first line of defense by decontaminating the wound; generation of multiple cytokines and growth factors allows them to do more than simply minimize the risk of infection^20,21^. In WT mice at day 4, immunohistochemical analysis of the active neutrophil marker myeloperoxidase (MPO) showed that neutrophils were recruited to the epidermal front of the wound area, while recruitment was reduced by epidermis-specific SIRT1 deletion (Fig. 3C and D). Furthermore, mast cells have also been demonstrated to be important for wound healing^20^. Toluidine blue staining showed that epidermal-specific deletion of SIRT1 reduced the recruitment of mast cells to granulation tissue and the adjacent wound area (Fig. 3E and F). Taken together, these data demonstrate that epidermis-specific SIRT1 deficiency inhibits the recruitment of neutrophils, macrophages and mast cells into the wound.

### Epidermis-specific deletion of SIRT1 inhibits the recruitment and activation of fibroblasts

**Figure 4:**
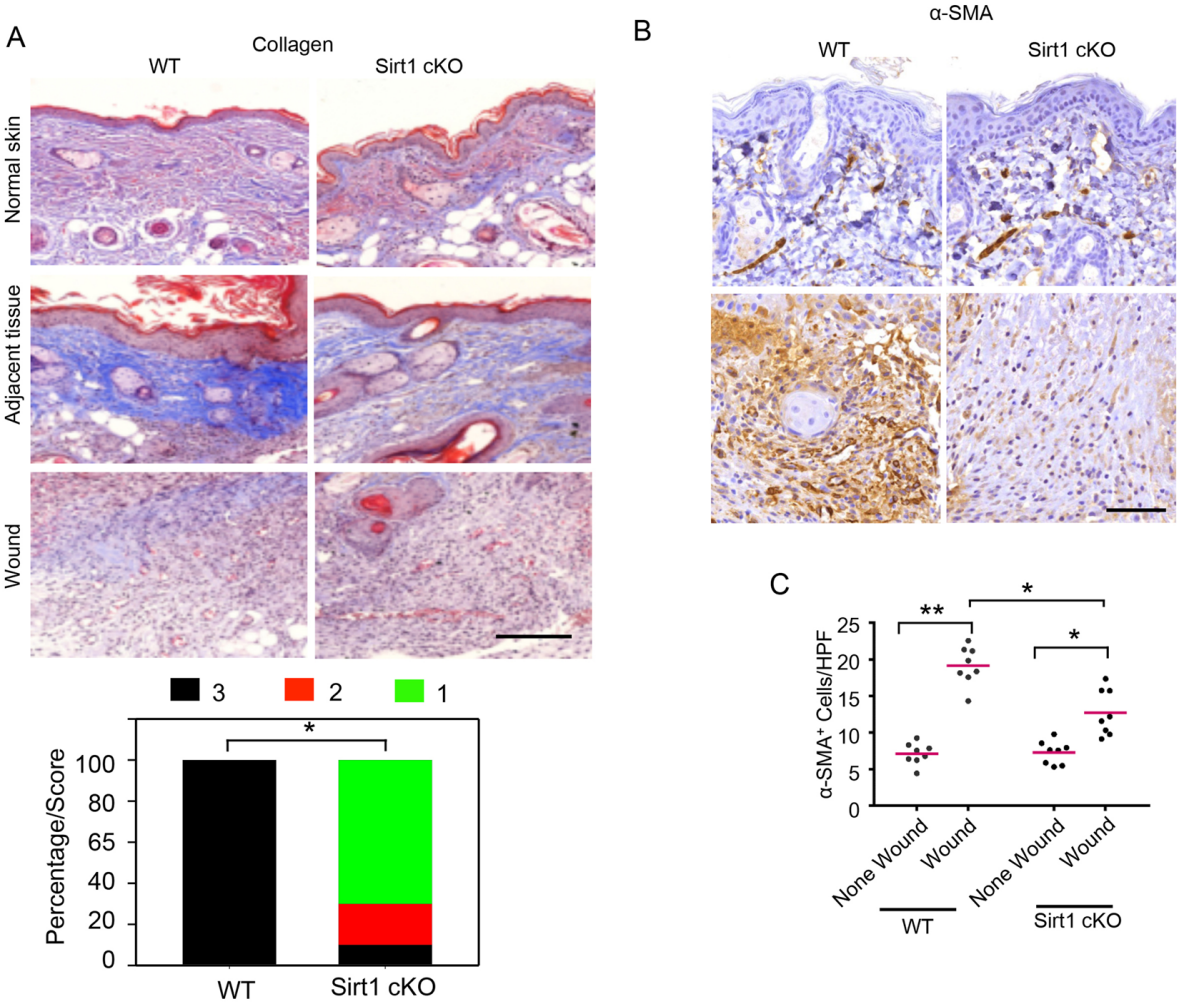
Epidermis-specific deletion of Sirt1 inhibits recruitment and activation of fibroblasts in the wound. (**A**) Mallory staining for collagen (blue) of normal and wound skin sections on day 6 in WT and Sirt1 cKO mice. Scale bars: 200 µm. Lower panel shows the percentage of adjacent dermis area (in stacked column format) for each score of collagen level. *P < 0.05; Mann-Whitney U test; between WT and Sirt1 cKO mice (n = 20). (**B**) Immunohistochemical staining of α-SMA in wound sections on day 6 in WT and Sirt1 cKO mice. Scale bar: 50 µm. N = 6 mice per group; n = 20 microscopic fields of skin sections per group. (**C**) Scatter plot and mean values of a-SMA^+^ cell number per high-power field (HPF; ×400 magnification) in wound sections on day 6 in WT and Sirt1 cKO mice (n = 8). *P < 0.05; **P < 0.01; between comparison groups.

The recruitment and activation of fibroblasts (myofibroblasts) has been demonstrated to be important for wound healing at least in part through regulating granulation tissue formation and angiogenesis as well as epidermis regeneration^22,23^. α-smooth muscle actin (α-SMA) is a marker of activated fibroblasts (myofibroblasts), which contributes to wound healing through regulating the secretion of extracellular matrix (ECM), matrix metalloproteinases, tissue inhibitors of metalloproteinases, and growth factors and cytokines important for wound healing^22,23^. WT mice had a greater number of α-SMA^+^ cells in the granulation tissue, in parallel with collagen deposition in the adjacent dermis and granulation tissue; in comparison, SIRT1 cKO mice showed accumulation of α-SMA^+^ cells in the adjacent dermis while it reduced the number of α-SMA^+^ cells in granulation tissue (Fig. 4A and B). These data demonstrated that epidermal-specific SIRT1 deficiency inhibits the recruitment and activation of fibroblasts in the wound.

### Epidermis-specific deletion of SIRT1 inhibits inflammatory cytokine production in the wound

**Figure 5:**
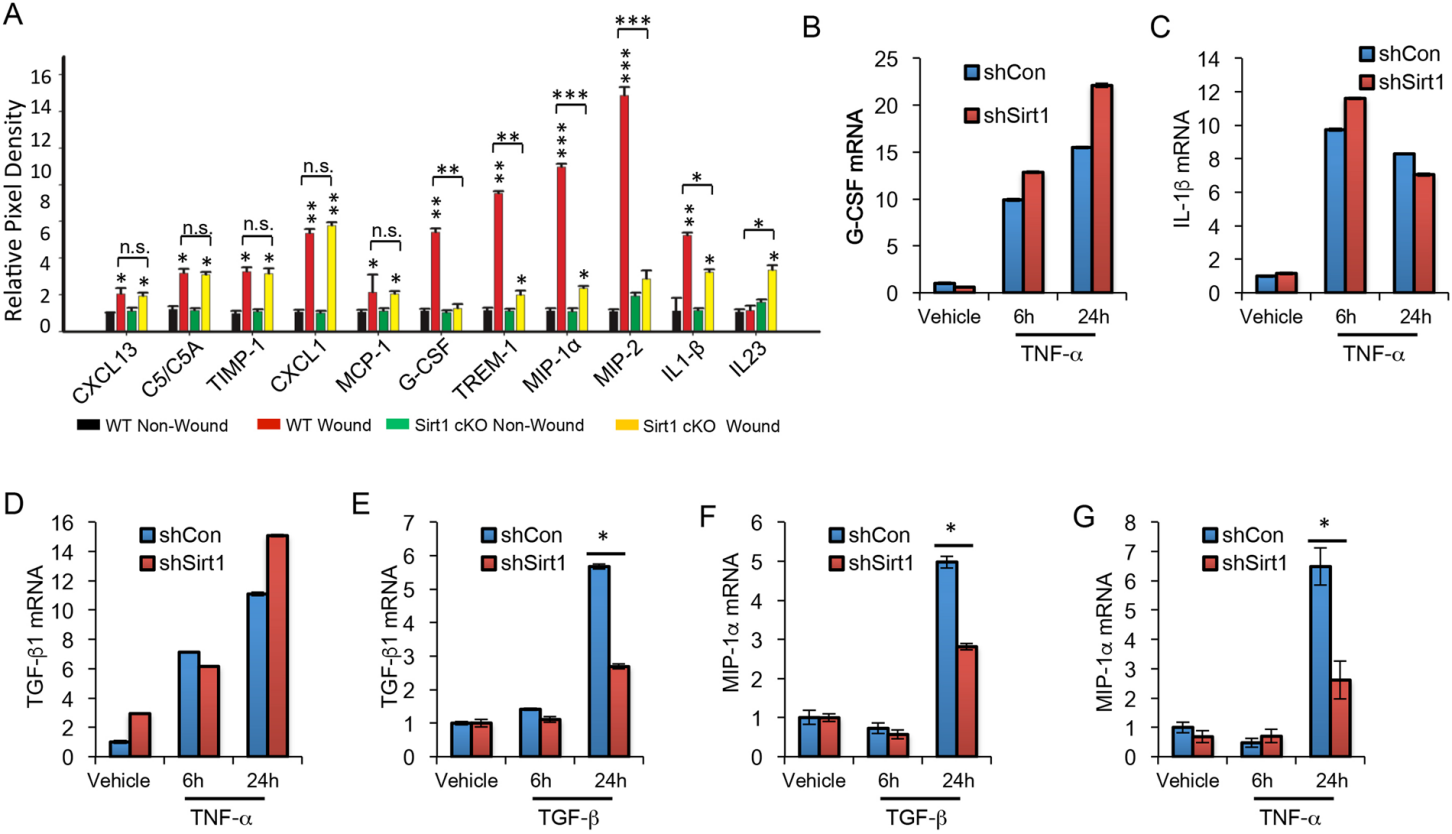
Epidermis-specific deletion of SIRT1 inhibits the production of inflammatory cytokines in the wound. (**A**) The relative mean pixel density (fold of WT Non-Wound) of cytokines was listed with a mouse cytokine array assay; *P < 0.05, **P < 0.01, ***P < 0.001, between comparison groups or with their corresponding non-wounding control groups (n = 3). (**B**–**G**) Real-time PCR analysis of G-CSF (**B**), IL-1β (**C**), TGF-β1 (**D**) with vehicle or TNF-α treatment, TGF-β1 (**E**) and MIP-1α (**F**) with vehicle or TGF-β treatment, and MIP-1α with vehicle or TNF-α treatment (**G**), in HaCaT cells stably infected with a lentiviral vector expressing control shRNA (shCon) or shRNA targeting SIRT1 (shSIRT1). *P < 0.05 between comparison groups (n = 3).

To determine the molecular mechanism by which SIRT1 regulates wound healing, we compared the difference in the levels of cytokines in wounds and adjacent areas between WT and epidermis-specific SIRT1 deletion mice, using a mouse cytokine array kit. The levels of CXCL13, C5/C5A, CXCL1, TREM-1, TIMP-1, G-CSF, MIP-1α, MIP-2 and IL-1β were increased in the wound and adjacent area, while the levels of TREM-1, G-CSF, MIP-1α, MIP-2 and IL-1β were reduced by epidermis-specific SIRT1 deletion (Fig. 5A and Supplemental Table S2). In the wound and adjacent area, epidermis-specific SIRT1 deletion increased the levels of IL23 (Figs 5A and S1). Previous studies have shown that double IL23 and IL12 knockout mice showed increased skin wound healing and influx of phagocytes and angiogenesis^24^. However, the role of IL23 alone remains to be investigated. To determine the mechanism by which SIRT1 regulates production of those cytokines, we measured the difference in gene expression in shCon and shSIRT1 HaCaT cells treated with TNF-α or TGF-β1. In TNF-α-treated HaCaT cells, SIRT1 knockdown increased the expression of G-CSF, IL-1β, and TGF-β1 at 6 h, or both 6 h and 24 h (Fig. 5B–D). However, In TGF-β1-treated HaCaT cells, SIRT1 knockdown inhibited the expression of TGF-β1 and MIP-1α (Fig. 5E and F). In addition, TNF-α also induced MIP-1α expression (Fig. 5G). MIP-1α is a chemokine that plays an important role in the influx of neutrophils and macrophages during early stage of wound healing^25^. These data demonstrated that SIRT1 inhibition reduces inflammatory cytokine production in the wound.

### SIRT1 is required for TGF-β induced epithelial-mesenchymal transition (EMT) in keratinocytes

**Figure 6:**
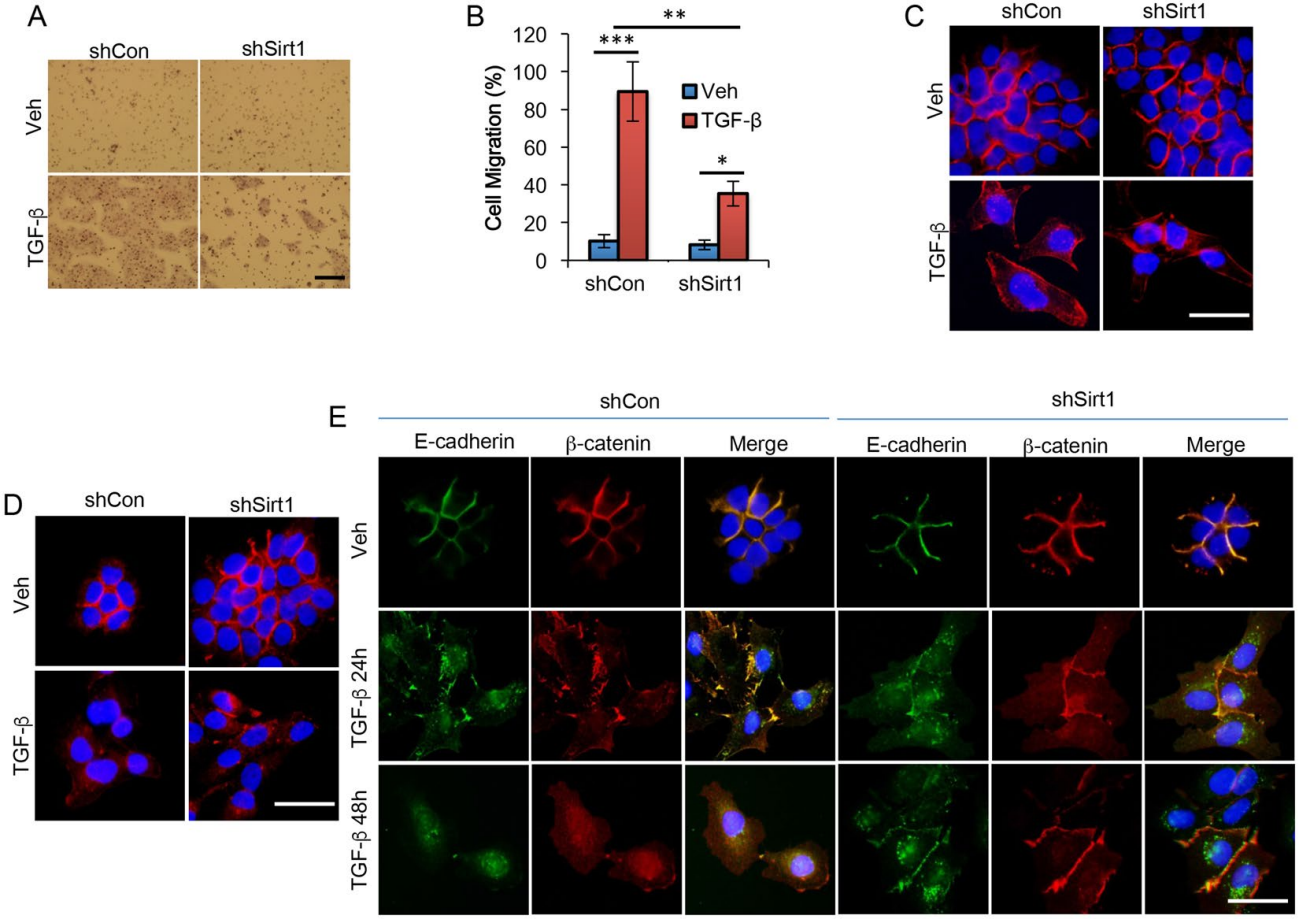
SIRT1 is required for TGF-β-induced migration and epithelial-mesenchymal transition (EMT) in HaCaT keratinocytes. (**A**) Transwell assay of HaCaT cells which were stably transfected with shCon or shSirt1 and treated with or without TGF-β (20 ng/ml) for 48 h in 1% FBS. Scale bar: 50 μm. (**B**) Quantification of A from three independent experiments; *P < 0.05; **P < 0.01; ***P < 0.001; between comparison groups (n = 3). (**C**) Immunohistochemical staining of F-actin in HaCaT cells stably transfected with shCon or shSirt1 and treated with or without TGF-β. Scale bar: 10 μm. (**D**) Immunohistochemical staining of ZO-1 in cells as in A. Scale bar: 10 μm. (**E**) Immunohistochemical staining of E-cadherin and β-catenin as in A for 24 or 48 h. Scale bar: 10 μm. DAPI (blue) was used as a nuclear counterstain.

The essence of cutaneous wound healing is to restore the epidermal barrier to the external environment during re-epithelialization. A key feature of re-epithelialization is the migration of keratinocytes in a manner that is reminiscent of EMT, which is orchestrated by injury signals such as the inflammatory cytokine TGF-β1. Therefore we investigated the role of SIRT1 in TGF-β1 induced EMT in keratinocytes. Using an *in vitro* transwell migration assay, we found that SIRT1 knockdown reduced TGF-β-induced directional migration of the human HaCaT cells (Fig. 6A and B). A classical feature of TGF-β1-induced EMT is the morphological change of epithelial cells to a mesenchymal spindle-shape that is accompanied by cytoskeletal reorganization through formation of stress fibers, which can be detected by phalloidin staining of filamentous actin (F-Actin). In control HaCaT cells (shCon), TGF-β1 induced the formation of actin stress fibers, which was reduced by SIRT1 knockdown (Fig. 6C). Epithelial cell junction proteins, such as the tight junction (TJ) protein zonula occludens (ZO-1), are critical in maintaining cell polarization and cell-cell contacts, and are often down-regulated or diffused away from the cell-cell contact during EMT^26^. Immunofluorescence analysis showed that TGF-β decreased ZO-1 levels at the cell-cell contacts in shCon cells, while SIRT1 knockdown partially prevented TGF-β-induced ZO-1 down-regulation in the cell-cell junction (Fig. 6D). In addition, TGF-β suppressed the colocalization of the adherens junction protein E-cadherin with β-catenin at the junction between adjacent cell membranes, while SIRT1 knockdown prevented such a change (Fig. 6E). These data demonstrate that SIRT1 is required for TGF-β-induced EMT.

### SIRT1 regulates

#### TGF-β signaling

**Figure 7:**
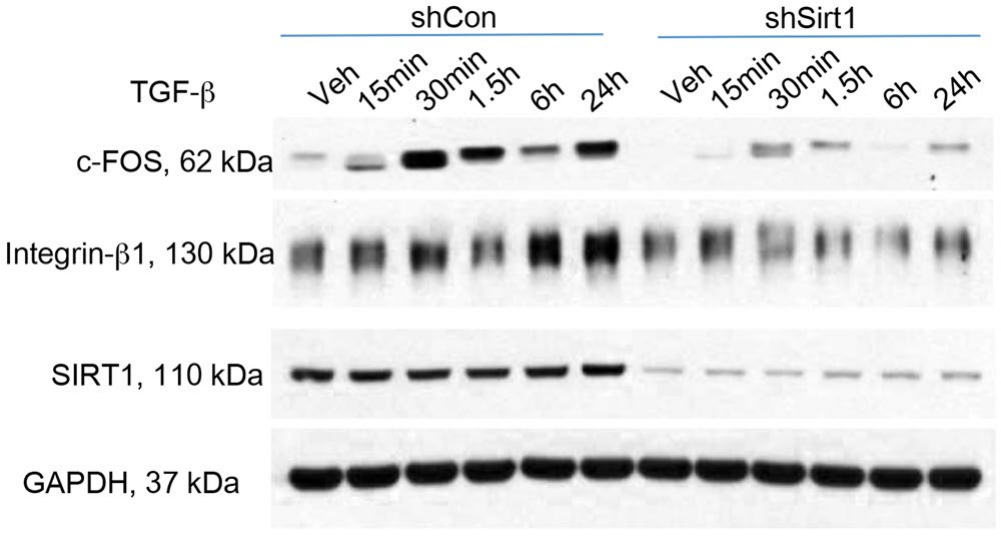
SIRT1 regulates TGF-β pathway in HaCaT keratinocytes. Immunoblot analysis of c-FOS, Integrin-β1, SIRT1, and GAPDH in HaCaT cells stably transfected with shCon or shSirt1 and treated with or without TGF-β (20 ng/ml) over the time course indicated.

To determine how SIRT1 regulates TGF-β signaling, HaCaT cells were treated with TGF-β for 48 h. TGF-β treatment induced the expression of c-FOS and Integrin-β1 (Fig. 7A), which was prevented by SIRT1 knockdown (Fig. 7A), indicating that SIRT1 inhibits TGF-β signaling These findings indicate that SIRT1 regulates TGF-β signaling.

## Discussion

SIRT1 is the proto member of the mammalian sirtuin family of proteins and plays multiple roles in aging and disease. In this study, using mice with epidermis-specific SIRT1 deletion, we show that SIRT1 is required for efficient wound healing. SIRT1 deficiency in the epidermis inhibits the regeneration of both the epidermis and the dermis stroma. SIRT1 loss altered the production of many cytokines, inhibited the recruitment of macrophages, neutrophils and mast cells, and reduced the angiogenesis in granulation tissue. In keratinocytes, SIRT1 knockdown inhibited EMT, cell migration, and TGF-β signaling, and increased oxidative stress. Our findings demonstrate a new function of SIRT1 in the epidermis in wound repair.

SIRT1 is known to regulate inflammation and stress response. SIRT1 has been shown to suppress the pro-inflammatory transcription factor NF-κB by deacetylating the p65 subunit and thus sensitizing cells to TNF-α-induced apoptosis^27^. We found that both TGF-β and TNF-α can induce MIP-1α expression (Fig. 5F and G). Although in mice MIP-1α knockout does not affect wound healing^28^, nevertheless SIRT1 does affect the production of multiple cytokines from the wound site. It is possible that more than one cytokine is involved, including IL-1β, a pro-inflammatory cytokine for the initial inflammation following wounding. Therefore it is possible that SIRT1 promotes keratinocyte inflammation during wounding. Future investigation is required to elucidate the role of inflammation and NF-κB in SIRT1 regulation of wound repair.

In addition, SIRT1 regulates cell migration. SIRT1 has been shown to interact with and activate TIAM1 (T-cell lymphoma invasion and metastasis 1) and Rac1 in tumor cells, in parallel with TIAM1 acetylation^29^. TIAM1 is the GEF (Guanine nucleotide exchange factor) that activates Rac1. In the context of wound healing, SIRT1 may have a similar role in cell migration of non-tumorigenic keratinocytes. (1) SIRT1 may regulate TGF-β-induced EMT through TIAM1 and Rac1, or (2) SIRT1 promotes TGF-β expression in response to TGF-β, providing a potential feed-forward mechanism. SIRT1 regulates the expression of c-FOS and integrin induced by TGF-β. TGF-β is shown to induce c-FOS expression in EL2 fibroblasts^30^, while C-FOS is required for TGF-β expression^31^. C-FOS binds with the AP1 site of the proximal promoter of TGF-β 1 to promote TGF-β expression and thus cell migration. TGF-β also induces the expression of beta1 integrin to promote adhesion of rat mesangial cells^32^. In our previous work, using microarray analysis, we compared the difference in gene expression between control and SIRT1-knockdown keratinocytes^16^. SIRT1 knockdown had no effect on the expression of TGF-β receptor genes^16^. Future investigation is required to elucidate the specific mechanism in the regulation of SIRT1 of TGF-β signaling.

SIRT1 may also promote epithelial regeneration through its function in survival or proliferation. SIRT1 is shown to inhibit TGF-β-induced apoptosis in glomerular mesangial cells^33^. Others and we have shown that SIRT1 is required for cell survival following UVB irradiation^15,34^. In addition, we found that SIRT1 inhibition decreased wound healing, while it moderately increased proliferation (Fig. 2), suggesting that SIRT1’s role in wound healing is a complex well-coordinated process and proliferation alone is not sufficient for wound repair. Possibly other functions of epidermal SIRT1, including regulating cell migration and inflammation, are the more important determinants for SIRT1’s role in wound healing. Future studies will elucidate the role of SIRT1’s pro-survival or proliferation function, and the coordination of these functions in wound healing.

SIRT1’s function in wound healing may be cell-type or wound healing stage-specific. In late stages of wound healing, SIRT1 also plays a role in suppressing scar formation by inhibiting TGF-β-induced fibroblast activation; it may be a promising target for preventing hypertrophic scar formation^35^. Fibroblast-specific SIRT1 deletion inhibits tissue fibrosis induced by bleomycin or TBRIact^36^. Future studies will elucidate the role of SIRT1 in keratinocytes in late stage wound healing such as scar formation. In addition, only one of the two SIRT1-transgenic mouse lines showed improved wound healing at an older age^18^. Therefore, the role of SIRT1 overexpression in different cell types in the healing of skin wounds remains unclear. It remains to be investigated whether, and how, epidermal SIRT1 overexpression regulates wound healing. In addition, the SIRT1 protein levels in the epidermis seem not be altered in wound skin as compared with control skin (Supplemental Fig. 1). Future investigation will elucidate how wounding regulates the SIRT1 pathways, including SIRT1 activity and targets.

In summary, our findings have demonstrated that epidermal SIRT1 is required for effective wound healing in mice. SIRT1 loss decreased keratinocyte migration and dermal stroma regeneration, while it increased oxidative stress in keratinocytes. Our results indicate a pivotal role of epidermal SIRT1 in wound healing through multiple mechanisms, and may open up new opportunities for future investigation of targeting epidermal SIRT1 to improve wound repair.

## Materials and Methods

### Cell culture

HaCaT (human keratinocytes, kindly provided by Dr. Norbert E. Fusenig, German Cancer Research Center, Heidelberg, Germany) and HEK-293T (human embryonic kidney cells) were maintained in a monolayer culture in 95% air/5% CO_2_ at 37 °C in Dulbecco’s modified Eagle’s medium (DMEM) supplemented with 10% fetal bovine serum (FBS), 100 units per mL penicillin and 100 μg/mL streptomycin (Invitrogen, Carlsbad, CA). For the TNF-α and TGF-β treatment, cells were washed with PBS twice and incubated with serum free DMEM medium overnight and then replaced with serum free DMEM supplemented with TNF-α (50 ng/ml, R&D Systems Inc., Minneapolis, MN) or TGF-β (50 ng/ml, R&D Systems Inc., Minneapolis, MN) for the indicated times.

### Animals and wound healing

All animal procedures were approved by the University of Chicago Institutional Animal Care and Use Committee. All experiments were performed in accordance with relevant guidelines and regulations. Wild-type (WT) and SIRT1 cKO mice in the SKH1 hairless background were generated as described previously^15,16^. Mice were housed 5 animals per cage, and there was no evidence of dorsal wounds caused by fighting. For skin wound healing assays, same sex littermates of ~10-week-old mice were anesthetized, and two 4 mm full-thickness excisional wounds were created on both sides of the dorsal midline (12). Tissue was collected at 2–8 days after wounding, and wound re-epithelialization was evaluated by histological analyses. Hyperproliferative epidermis (HE) was identified by hematoxylin and eosin staining, and the length of HE that extended into the wounds was measured and quantified.

### Plasmid transfection

Knockdown of Sirt1 in HaCaT cells was performed using lentiviral vectors, with HEK293T cells used as packaging cells for the lentiviral system. pLKO.1 vectors, pLKO.1 shSirt1 (Sigma-Aldrich, St. Louis, MO) and packaging mix (psPAX2 and pMG2.G) were transfected into HEK293T cells using X-tremeGENE HP (Roche, Basel, Switzerland). Two days after transfection, the supernatants were collected and diluted with the same volume of fresh complete Dulbecco’s modified Eagle’s medium, and Polybrene was added to the infection medium at 8 μg/ml. The infection mixtures were added to cell cultures seeded on a 6-well plate, incubated overnight, and then replaced with fresh complete Dulbecco’s modified Eagle’s medium containing 8 μg/ml puromycin. The puromycin-resistant cells were isolated and propagated for use in the experiments.

### Immunofluorescence

Immunofluorescence in tissues was performed as described previously^16,37–39^. Briefly, the skin tissues were embedded, fixed, permeabilized, incubated with primary mouse anti-K5 (1:200; Progen Biotechnik, Heidelberg, Germany), rabbit anti-Filaggrin (1:1000; Abcam, Cambridge, MA), rabbit anti-E-cadherin (1:500; Cell Signaling, Danvers, MA), mouse anti-β-catenin (Cell Signaling, Danvers, MA), rabbit anti-F-actin (Cell Signaling, Danvers, MA) and rabbit anti-ZO-1(Cell Signaling, Danvers, MA), and then incubated with Alexa Fluor 488 F (ab’) 2 fragments of goat anti-guinea pig IgG antibodies and Alexa Fluor 568 of goat anti-rabbit IgG antibodies (Invitrogen, Carlsbad, CA). The cells were then fixed in Prolong Gold Antifade with DAPI (Invitrogen, Carlsbad, CA) to visualize the cell nuclei, and observed under a fluorescence microscope (OlympusIX71, Japan) with a peak excitation wavelength of 340 nm.

### Western Blotting

Western blotting was performed as described previously^40^. Antibodies used were as follows: Sirt1 and GAPDH were obtained from Santa Cruz (Dallas, TX); p-p38, c-Fos, p-JNK, p-Smad3 and Integrin-beta1 were obtained from Cell Signaling (Danvers, MA).

### Real-time PCR

Quantitative real-time PCR assays were performed using a CFX Connect real-time system (Bio-Rad, Hercules, CA) using Bio-Rad iQ SYBR Green Supermix^14,37,41^. The threshold cycle number (CQ) for each sample was determined in triplicate and normalized against GAPDH as described previously^41^. Amplification primers are listed in Supplemental Table S1.

### Toluidine blue staining for mast cells

Toluidine blue staining for mast cells was performed according to the manufacturer’s protocol (IW-3013; NovaUltra, Waltham, MA) as described previously^16^.

### *In vitro* cell proliferation assay

Cell proliferation of HaCaT cells stably transfected with shCon and shSirt1 was analyzed using the MTS assay (Promega, Madison, WI, USA) according to the manufacturer’s instructions as in our recent studies.

### Masson trichrome staining

Masson trichrome staining for collagen fibers of the dermis was performed according to the manufacturer’s protocol (Procedure No. HT15, Sigma-Aldrich, St. Louis, MO). Collagen content was analyzed double blindly by two independent investigators with a scale of 1 (weak), 2 (moderate), and 3 (strong).

### Cytokine array

A mouse cytokine array was performed using the mouse Cytokine Array Panel A (#ARY006; R&D Systems) following the manufacturer’s instructions. 300 µg tissue lysates were incubated with a separate array precoated with 40 cytokine/chemokine duplicate antibodies and labeled with streptavidin-HRP-conjugated secondary antibody and developed using chemiluminescence. The intensity of the selected dots was analyzed using ImageJ (NIH) software. Duplicates were averaged and the background subtracted to calculate the mean pixel density for each protein. Names of cytokines are listed in Supplemental Table S2.

### Histological and Immunohistochemical analysis

Hematoxylin and eosin (H&E) staining and F4/80, CD31, ki67, and αSMA and MPO (Myeloperoxidase) immunohistochemical analysis were performed by the Immunohistochemistry core facility at the University of Chicago.

### Transwell assay

The transwell filters (8 μm pore size Millipore MultiScreen-MIC plates) were used to analyze the migration activity of HaCaT cells according to the manufacturer’s instructions.

### Statistical analyses

Statistical analyses were carried out using Prism 6 (GraphPad software, San Diego, CA, USA) as described previously^37^. Data are reported as mean ± S.D. Results between two independent groups were determined by unaired Student’s t-test or Mann-Whitney U test unless otherwise stated. P values less than 0.05 were considered significant.

### Data availability

All data generated or analyzed during this study are included in this published article.

## Electronic supplementary material

Supplemental information
