# Supplementary material for: Epidermal SIRT1 regulates inflammation, cell migration, and wound healing
Source: Sci Rep. 2017 Oct 26;7:14110. doi: 10.1038/s41598-017-14371-3 (PMC5658409; doi:10.1038/s41598-017-14371-3)
Supplement: Supplementary file 1 — Supplemental information [file 41598_2017_14371_MOESM1_ESM.pdf]

Epidermal SIRT1 regulates inflammation, cell migration, and wound healing

Lei Qiang[1,2*], Ashley Sample[1,3], Han Liu[4], Xiaoyang Wu[3,4], and Yu-Ying He[1,3*]

Supplemental Figure 1

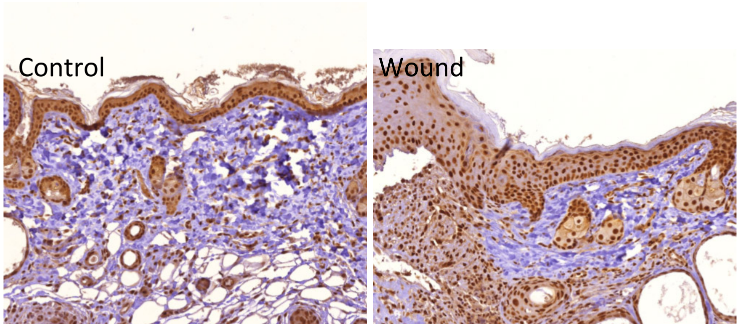

Supplemental figure 1. Immunohistochemical analysis of the SIRT1 protein level in unwounded (control) and wounded skin (Wound).

Supplemental Figure 2

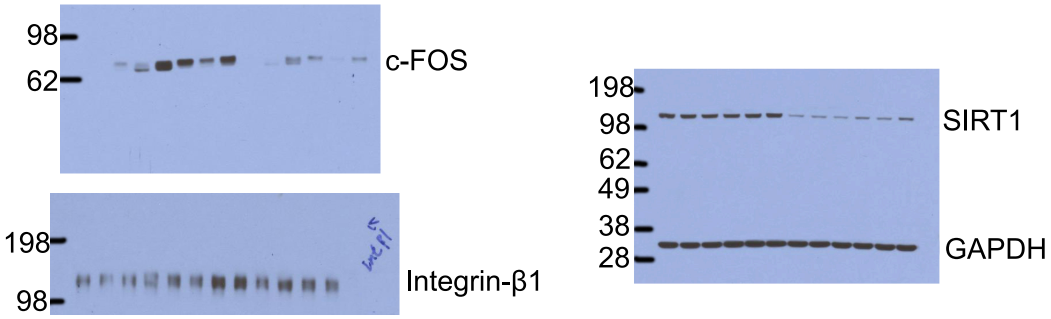

Supplemental figure 2. Gel image of the immunoblot analysis shown in Figure 7, marked with molecular weight marker and protein name.

Supplementary Table S1. Sequences for RT-qPCR primers.

Gene Name	Size	Forward Primer	Reverse Primer
CCL1	90	5'-AATACCAGCTCCATCTGCTCCAA-3'	5'-GAACCCATCCAACTGTGTCCAAG-3'
CXCL1	108	5'-AACCGAAGTCATAGCCACAC-3'	5'-GTTGGATTTGTCACTGTTCAGC-3'
G-CSF	142	5'-GAGTTGGGTCCCACCTTG-3'	5'-TGGAAAGCAGAGGCGAAG-3'
GM-CSF	77	5'-CACTGCTGCTGAGATGAATGAAA-3'	5'-GTCTGTAGGCAGGTCGGCTC-3'
IL1-B	73	5'-GCTTGGTGATGTCTGGTCCAT-3'	5'-CACCACTTGTTGCTCCATATCCT-3'
TGF-B	136	5'-CAGCAACAATTCCTGGCGATA5-3'	5'-AAGGCGAAAGCCCTCAATTT-3'
MIP-1a	106	5'-CTGCATCACTTGCTGCTGACA-3'	5'-CACTGGCTGCTCGTCTCAAAG-3'
GAPDH	82	5'-AATCCCATCACCATCTTCCA-3'	5'-TGGACTCCACGACGTACTCA-3'

* Primers were designed based on human CDS of targets found in NCBI database. All primers were validated using human universal cDNA.

Supplementary Table S2. Appendix for the Mouse Cytokine Array coordinates (R&D, ARY006)

Coordinate	Target/Control	Alternate Nomenclature
A1,A2	Reference Spot	N/A
A23,A24	Reference Spot	N/A
B1,B2	BLC	CXCL13/BCA-1
B3,B4	C5/C5α	Complement Component 5/5α
B5,B6	G-CSF	N/A
B7,B8	GM-CSF	N/A
B9,B10	I-309	CCL1/TCA-3
B11,B12	Eotaxin	CCL11
B13,B14	sICAM-1	Cd54
B15,B16	IFN-γ	Type II IFN
B17,B18	IL-1α	IL-1F1
B19,B20	IL-1β	IL-1F2
B21,B22	IL-1ra	IL-1F3
B23,B24	IL-2	N/A
C1,C2	IL-3	N/A
C3,C4	IL-4	N/A
C5,C6	IL-5	N/A
C7,C8	IL-6	N/A
C9,C10	IL-7	N/A
C11,C12	IL-10	N/A
C13,C14	IL-13	N/A
C15,C16	IL-12p70	N/A
C17,C18	IL-16	N/A
C19,C20	IL-17	N/A
C21,C22	IL-23	N/A
C23,C24	IL-27	N/A
D1,D2	IP-10	CXCL10/CRG-2
D3,D4	I-TAC	CXCL11
D5,D6	KC	CXCL1
D7,D8	M-CSF	N/A
D9,D10	JE	CCL2/MCP-1
D11,D12	MCP-5	CCL12
D13,D14	MIG	CXCL9
D15,D16	MIP-1α	CCL3
D17,D18	MIP-1β	CCL4
D19,D20	MIP-2	CXCL2
D21,D22	RANTES	CCL5
D23,D24	SDF-1	CXCL12
E1,E2	TRAC	CCL17

E3,E4	TIMP-1	N/A
E5,E6	TNF-α	N/A
E7,E8	TREM-1	N/A
F1,F2	Reference Spot	N/A
F23,F24	PBS (Negative Control)	N/A
